# Supplementary material for: Increased activity of procoagulant factors in patients with small cell lung cancer
Source: PLoS One. 2021 Jul 21;16(7):e0253613. doi: 10.1371/journal.pone.0253613 (PMC8294523; doi:10.1371/journal.pone.0253613)
Supplement: S1 Table — (PDF) [file pone.0253613.s001.pdf]

Baseline Biochemistry

	Sodium (mmol/l)	Potassim (mmol/l)	Calcium (mmol/l)	Creatinine (µmol/l)
1	131	4.1	2.59	49
2	140	4.2	2.38	68
3	138	4.1	2.45	53
4	135	3.1	2.53	53
5	138	4	2.6	50
6	141	3.9	2.48	66
7	145	3.5	2.58	76
8	140	4	2.49	69
9	127	3.1	2.43	42
10	139	4.5	2.48	82
11	136	4.1	2.51	31
12	138	4.7	2.65	70
13	139	3.9	2.53	79
14	138	3.7	2.54	61
15	144	4	2.48	96
16	133	3.9	2.37	75
17	128	4.2	2.4	65
18	145	4	2.34	103
19	143	4	2.55	78
20	140	4.4	2.52	63
21	134	4.4	2.57	195
22	138	3.8	2.49	33
23	142	3.5	2.54	74
24	140	3.8	2.46	53
25	140	4.4	2.36	57
26	136	4.3	2.51	70
27	139	4.1	2.44	61
28	140	4.2	2.42	74
29	138	4.1	2.57	43
30	140	4	2.53	53
31	138	3.9	2.5	70
32	141	4.1	2.52	93
33	135	3.4	2.49	84
34	136	4.4	2.24	35
35	139	3.7	2.58	59
36	137	4.9	2.55	97
37	138	3.1	2.29	41
38	138.1	4.0	2.5	68.4

Baseline Blood counts

Albumin (g/l)		Sodium (mmol/l)	Potassim (mmol/l)
33	1	333	12.7
33	2	440	8.5
36	3	349	9.3
39	4	373	9.3
30	5	203	10.2
32	6	612	14.1
38	7	217	8.8
35	8	273	8.1
38	9	359	7.4
37	10	426	9.1
35	11	406	7.2
32	12	363	11.1
31	13	378	9.5
43	14	364	9
29	15	332	8.4
36	16	432	10.1
40	17	442	12
40	18	559	11.4
37	19	315	8.5
19	20	501	12.7
30	21	348	8.6
22	22	307	5.6
43	23	441	14.9
39	24	186	10.4
39	25	286	9.3
32	26	133	8.2
38	27	451	10.7
37	28	238	10.6
22	29	509	8.6
41	30	225	8
37	31	243	7.9
37	32	460	8.8
36	33	484	10.7
32	34	326	11.7
27	35	371	7.4
31	36	191	5.4
33	37	277	4.9
34.6	38	355	9.4
