# Supplementary figures and images for: Increased activity of procoagulant factors in patients with small cell lung cancer
Source: PLoS One. 2021 Jul 21;16(7):e0253613. doi: 10.1371/journal.pone.0253613 (PMC8294523; doi:10.1371/journal.pone.0253613)

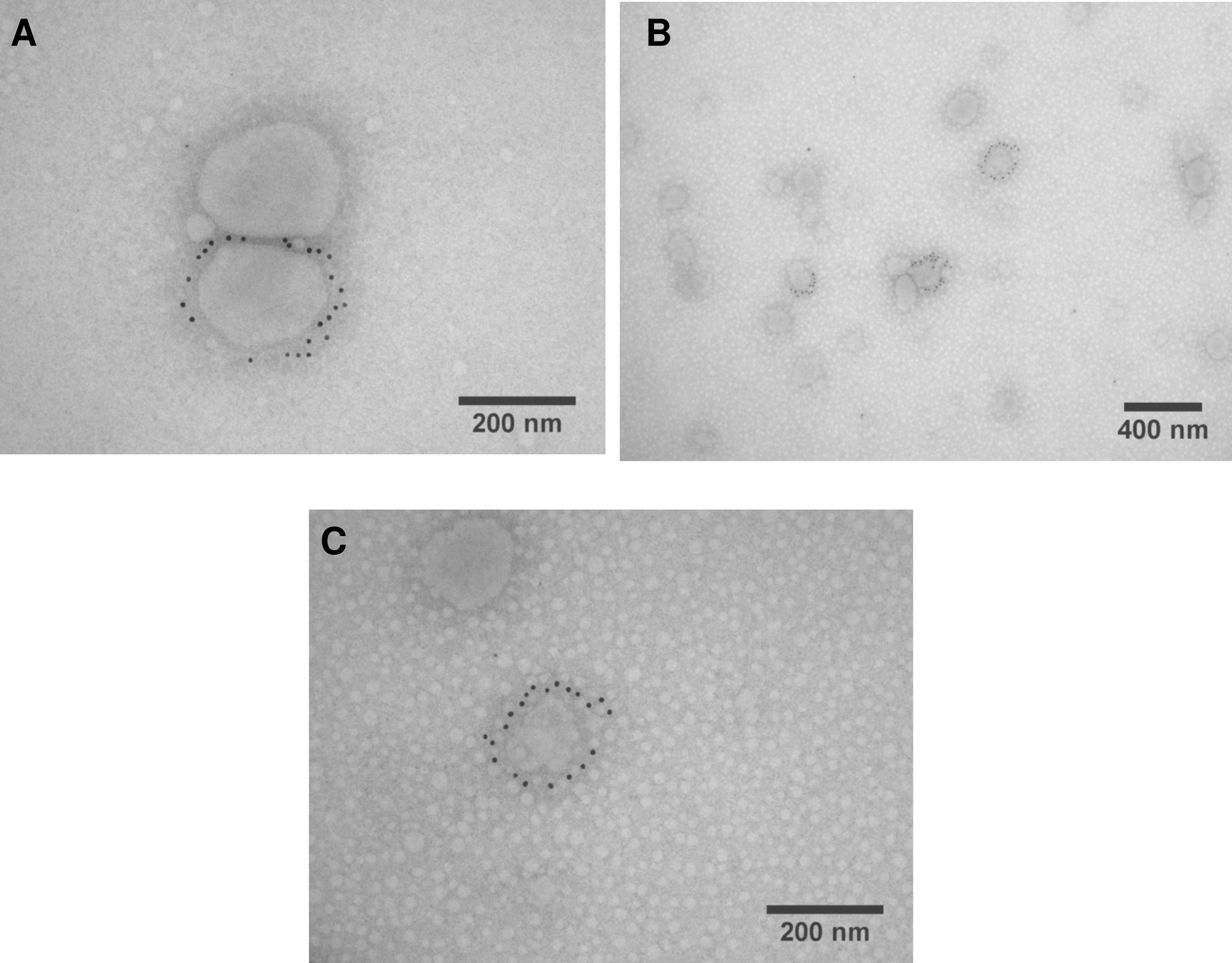

Supplement: S1 Fig — A) 20K pel CD9 positive vesicles. B) Both CD9 positive and negative vesicles isolated for the 100K pel. C) 100K pel CD9 positive vesicle (S2 Fig). (TIF) [file pone.0253613.s002.tif]

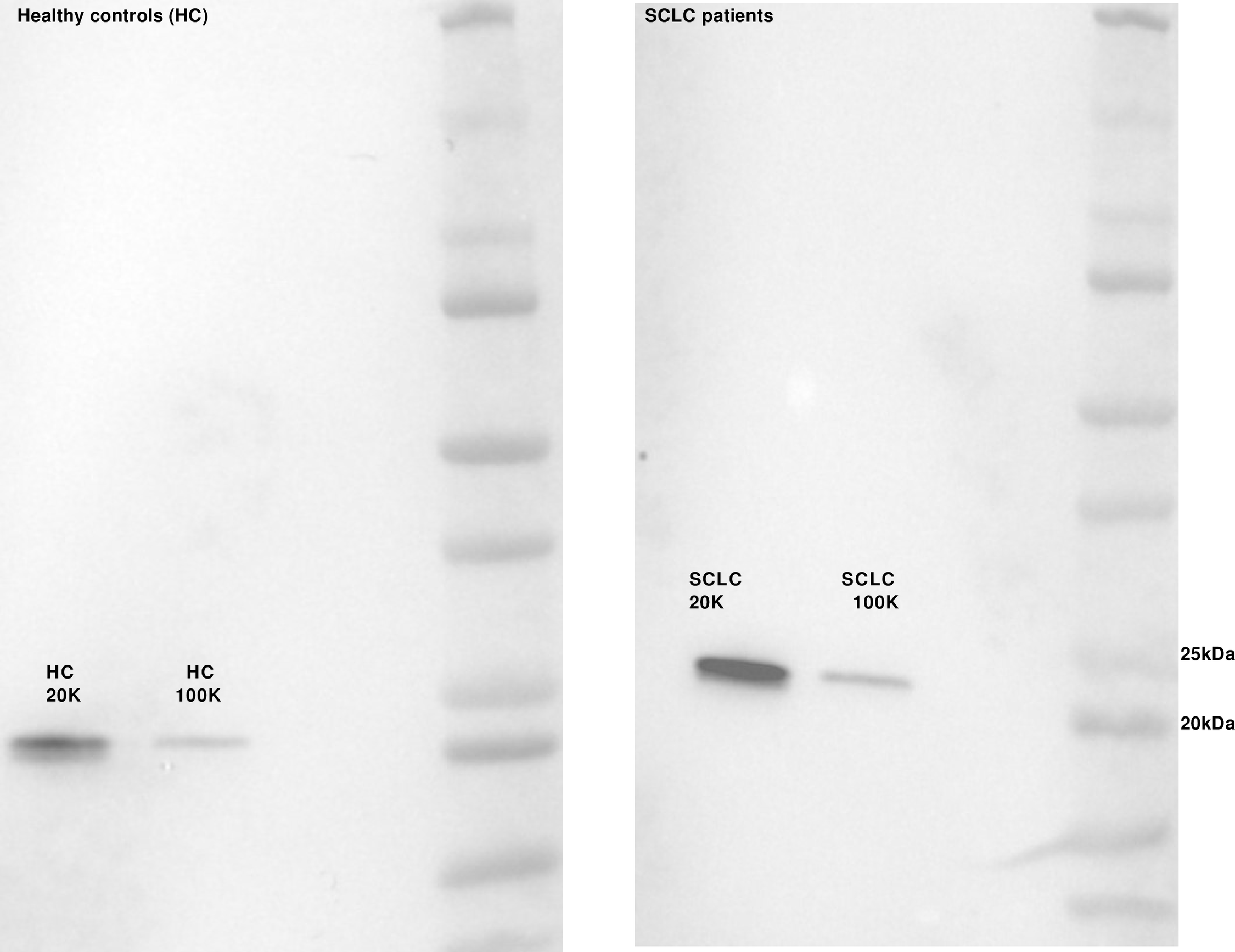

Supplement: S2 Fig — (TIF) [file pone.0253613.s003.tif]
