# Supplementary material for: Increased activity of procoagulant factors in patients with small cell lung cancer
Source: PLoS One. 2021 Jul 21;16(7):e0253613. doi: 10.1371/journal.pone.0253613 (PMC8294523; doi:10.1371/journal.pone.0253613)
Supplement: Attachment — Submitted filename: Response to reviewers.docx [file pone.0253613.s004.docx]

Dear Editor and Reviewers,

We thank you for the time and effort in reviewing our manuscript. We believe that the advised revisions and comments has heightened the scientific content and coherence of our article. In the document below, we have addressed and responded to the reviewer’s and editors’ comments - point-by-point. Throughout the manuscript, revisions are clarified using track changes mode.

In accordance to point 4, we have also included, under Supporting Information files, the original and uncropped Immunoelectron microscopy (IEM) images (see S2_raw_IEM images.pdf) and western blots (see S3_raw_WB.pdf).

In accordance to point 5, we have removed the ‘financial disclosure statement’ from the manuscript.

We would like to state under Financial disclosure statement that:

1. “This work was funded by grants from the Danish Research Council for Independent Research, 4183-00268), https://ufm.dk/ (S.R.K.); and the Obel Family Foundation, 26145, http://www.european-funding-guide.eu/scholarship/7862-obel-family-foundation

(S.R.K.).

2. “The funders had no role in study design, data collection and analysis, decision to

publish, or preparation of the manuscript.”

On behalf of the authors, we would like to express our appreciation for reviewing our manuscript.

Best regards,

Shona Pedersen, Senior Scientist, Associate Professor

Dep. of Clinical Biochemistry and Clinical Medicine

Aalborg University hospital, Aalborg University

Aalborg, Denmark

PONE-D-21-04828
Increased activity of procoagulant factors in patients with small cell lung cancer
PLOS ONE

Dear Dr. Pedersen,

Thank you for submitting your manuscript to PLOS ONE. After careful consideration, we feel that it has merit but does not fully meet PLOS ONE’s publication criteria as it currently stands. Therefore, we invite you to submit a revised version of the manuscript that addresses the points raised during the review process.
Thank you for the opportunity to submit a revised paper.

Please submit your revised manuscript by Apr 25 2021 11:59PM. If you will need more time than this to complete your revisions, please reply to this message or contact the journal office at [plosone@plos.org](mailto:plosone@plos.org). When you're ready to submit your revision, log on to <https://www.editorialmanager.com/pone/> and select the 'Submissions Needing Revision' folder to locate your manuscript file.

Please include the following items when submitting your revised manuscript:

- A rebuttal letter that responds to each point raised by the academic editor and reviewer(s). You should upload this letter as a separate file labeled 'Response to Reviewers'.
- A marked-up copy of your manuscript that highlights changes made to the original version. You should upload this as a separate file labeled 'Revised Manuscript with Track Changes'.
- An unmarked version of your revised paper without tracked changes. You should upload this as a separate file labeled 'Manuscript'.

If you would like to make changes to your financial disclosure, please include your updated statement in your cover letter. Guidelines for resubmitting your figure files are available below the reviewer comments at the end of this letter.

If applicable, we recommend that you deposit your laboratory protocols in protocols.io to enhance the reproducibility of your results. Protocols.io assigns your protocol its own identifier (DOI) so that it can be cited independently in the future. For instructions see: [http://journals.plos.org/plosone/s/submission-guidelines#loc-laboratory-protocols](https://eur03.safelinks.protection.outlook.com/?url=http%3A%2F%2Fjournals.plos.org%2Fplosone%2Fs%2Fsubmission-guidelines%23loc-laboratory-protocols&data=04%7C01%7C%7C22b8cd8b1d524d49f8f208d8e4d8a5f8%7C5968b90c51a64f088b4750ffffbe2e4f%7C0%7C0%7C637510967656768078%7CUnknown%7CTWFpbGZsb3d8eyJWIjoiMC4wLjAwMDAiLCJQIjoiV2luMzIiLCJBTiI6Ik1haWwiLCJXVCI6Mn0%3D%7C1000&sdata=J7yOsc0%2FJ94snLcu8eFohfZd8M3SavSPo21mCep%2BXns%3D&reserved=0)

We look forward to receiving your revised manuscript.

Kind regards,

Hugo ten Cate, MD, PhD
Academic Editor
PLOS ONE

Journal Requirements:

When submitting your revision, we need you to address these additional requirements.

1. Please ensure that your manuscript meets PLOS ONE's style requirements, including those for file naming. The PLOS ONE style templates can be found at

<https://journals.plos.org/plosone/s/file?id=wjVg/PLOSOne_formatting_sample_main_body.pdf> and

[https://journals.plos.org/plosone/s/file?id=ba62/PLOSOne_formatting_sample_title_authors_affiliations.pdf](about:blank)

2. Thank you for including your ethics statement:  "The study was approved by the regional Ethical Committee (N-20140055) and in accordance with the Declaration of Helsinki. ".

Please amend your current ethics statement to include the full name of the ethics committee/institutional review board(s) that approved your specific study. Thank for pointing this out. Our ethics statement has been revised to “Regional ethics committee for Northern Jutland” and this statement has also been included at the beginning of the Methods section.

For additional information about PLOS ONE ethical requirements for human subjects research, please refer to <http://journals.plos.org/plosone/s/submission-guidelines#loc-human-subjects-research>.

3. We note that you have included the phrase “data not shown” in your manuscript. Unfortunately, this does not meet our data sharing requirements. PLOS does not permit references to inaccessible data. We require that authors provide all relevant data within the paper, Supporting Information files, or in an acceptable, public repository. Please add a citation to support this phrase or upload the data that corresponds with these findings to a stable repository (such as Figshare or Dryad) and provide and URLs, DOIs, or accession numbers that may be used to access these data. Or, if the data are not a core part of the research being presented in your study, we ask that you remove the phrase that refers to these data.

We apologize for this fault. We have included the missing data under Supporting Information files, S1 Table. SCLC Baseline biochemistry and blood count data.

4. PLOS ONE now requires that authors provide the original uncropped and unadjusted images underlying all blot or gel results reported in a submission’s figures or Supporting Information files. This policy and the journal’s other requirements for blot/gel reporting and figure preparation are described in detail at <https://journals.plos.org/plosone/s/figures#loc-blot-and-gel-reporting-requirements> and <https://journals.plos.org/plosone/s/figures#loc-preparing-figures-from-image-files>. When you submit your revised manuscript, please ensure that your figures adhere fully to these guidelines and provide the original underlying images for all blot or gel data reported in your submission. See the following link for instructions on providing the original image data: <https://journals.plos.org/plosone/s/figures#loc-original-images-for-blots-and-gels>.

In your cover letter, please note whether your blot/gel image data are in Supporting Information or posted at a public data repository, provide the repository URL if relevant, and provide specific details as to which raw blot/gel images, if any, are not available. Email us at [plosone@plos.org](mailto:plosone@plos.org) if you have any questions.

5. Thank you for stating the following in the 'Financial disclosure statement' Section of your manuscript:

"This work was funded by grants from the Danish Research Council for 512 Independent Research (4183-
513 00268) and the Obel Family Foundation (26145)."

We note that you have provided funding information that is not currently declared in your Funding Statement. However, funding information should not appear in the Acknowledgments section or other areas of your manuscript. We will only publish funding information present in the Funding Statement section of the online submission form.

Please remove any funding-related text from the manuscript and let us know how you would like to update your Funding Statement. Currently, your Funding Statement reads as follows: We apologize for this error. We have deleted the funding-related text from the manuscript.

"The funders had no role in study design, data collection and analysis, decision to publish, or preparation of the manuscript."

Please include your amended statements within your cover letter; we will change the online submission form on your behalf.

Additional Editor Comments:

The expert reviewers raise a fairly large number of issues that need to be addressed and a major revision of this manuscript is therefore required before further decisions as to potential publication can be made.


[Note: HTML markup is below. Please do not edit.]

Reviewers' comments:

Reviewer's Responses to Questions

**Comments to the Author**

1. Is the manuscript technically sound, and do the data support the conclusions?

The manuscript must describe a technically sound piece of scientific research with data that supports the conclusions. Experiments must have been conducted rigorously, with appropriate controls, replication, and sample sizes. The conclusions must be drawn appropriately based on the data presented.

Reviewer #1: No

Reviewer #2: Yes

2. Has the statistical analysis been performed appropriately and rigorously?

Reviewer #1: Yes

Reviewer #2: Yes

3. Have the authors made all data underlying the findings in their manuscript fully available?

The [PLOS Data policy](https://eur03.safelinks.protection.outlook.com/?url=http%3A%2F%2Fwww.plosone.org%2Fstatic%2Fpolicies.action%23sharing&data=04%7C01%7C%7C22b8cd8b1d524d49f8f208d8e4d8a5f8%7C5968b90c51a64f088b4750ffffbe2e4f%7C0%7C0%7C637510967656778071%7CUnknown%7CTWFpbGZsb3d8eyJWIjoiMC4wLjAwMDAiLCJQIjoiV2luMzIiLCJBTiI6Ik1haWwiLCJXVCI6Mn0%3D%7C1000&sdata=KMXrF4qd%2FmzO2QFQA40ZwZ7OkxUQfanaRsFhnhrMfwI%3D&reserved=0) requires authors to make all data underlying the findings described in their manuscript fully available without restriction, with rare exception (please refer to the Data Availability Statement in the manuscript PDF file). The data should be provided as part of the manuscript or its supporting information, or deposited to a public repository. For example, in addition to summary statistics, the data points behind means, medians and variance measures should be available. If there are restrictions on publicly sharing data—e.g. participant privacy or use of data from a third party—those must be specified.

Reviewer #1: Yes

Reviewer #2: Yes

4. Is the manuscript presented in an intelligible fashion and written in standard English?

PLOS ONE does not copyedit accepted manuscripts, so the language in submitted articles must be clear, correct, and unambiguous. Any typographical or grammatical errors should be corrected at revision, so please note any specific errors here.

Reviewer #1: Yes

Reviewer #2: Yes

5. Review Comments to the Author

Please use the space provided to explain your answers to the questions above. You may also include additional comments for the author, including concerns about dual publication, research ethics, or publication ethics. (Please upload your review as an attachment if it exceeds 20,000 characters)

We thank the reviewers for their time reviewing this paper and for their many insightful comments. We have revised the paper according to theses comments. Below are our responses to the specific points:

Reviewer #1:

1/ Page 5. “which is considered to be the main initiator of coagulation”. TF is the main initiator of coagulation. We agree, and this has now been revised in page 5

2/ Page 5. Ref 11 is quite old. A more recent review on TF that discusses its role in thrombosis in PMID:29437578. Grover SP, Mackman N. Tissue Factor: An Essential Mediator of Hemostasis and Trigger of Thrombosis. Arterioscler Thromb Vasc Biol. United States; 2018;38(4):709–25-PMID:29437578, has now been replaced as ref **11** (page 5).

3/ Page 5. Ref 7,12, 13. PMID:23798713 should be added when discussing TF+ EVs and thrombosis in cancer. Geddings JE, Mackman N. Tumor-derived tissue factor – positive microparticles and venous thrombosis in cancer patients. Blood. 2013;122(11):1873–80-PMID: 23798713, has now been added as ref **14 (**page 5).

4/ Page 5. Ref 8. Two other studies have reported an association between EV TF activity and survival in cancer patients PMID:23856554; PMID:29539580. Bharthuar A, Khorana A a, Hutson A, Wang J-G, Key NS, Mackman N, et al. Circulating microparticle tissue factor, thromboembolism and survival in pancreaticobiliary cancers. Thromb Res. Elsevier B.V.; 2013;132(2):180–4- PMID:23856554 and Hisada Y, Thålin C, Lundström S, Wallén H, Mackman N. Comparison of microvesicle tissue factor activity in non-cancer severely ill patients and cancer patients. Thromb Res. 2018;165:1–5- PMID: 29539580, has now been included as reference **15** and **16**, respectively (page 5).

5/ Page 5 FVIII and risk of cancer-associated thrombosis. PMID:31945589 should be added. Moik F, Posch F, Grilz E, Scheithauer W, Pabinger I, Prager G, et al. Haemostatic biomarkers for prognosis and prediction of therapy response in patients with metastatic colorectal cancer. Thromb Res. 2020;187:9–17. ref **22 (page 5).**

6/ Page 5 “Thus, an increase in levels of EVs containing TF and PPL may play a crucial role for the risk of VTEs in cancer patients”. Additional references should be added. PMID:23798713; PMID:28807983.
Hisada Y, Mackman N. Cancer-associated pathways and biomarkers of venous thrombosis. Blood. 2017;130(13):1499–506. -PMID: 23798713 and PMID:28807983, has now been added as ref **25 and 14** (page 5).

7/ Page 5. More recent references describing the role of NETs in cancer are PMID:24590420; PMID:31315434. Demers M, Wagner DD. NETosis: a new factor in tumor progression and cancer-associated thrombosis. Semin Thromb Hemost. 2014;40(3):277–83- PMID: 24590420 and Thålin C, Hisada Y, Lundström S, Mackman N, Wallén H. Neutrophil Extracellular Traps: Villains and Targets in Arterial, Venous, and Cancer-Associated Thrombosis. Arterioscler Thromb Vasc Biol. 2019;39(9):1724–38- PMID: 31315434, has now been added as ref **27 and 28** (page 5).

8/ Page 6. There are other sources of cfDNA in plasma. cfDNA is not a good marker of NETs. This section should be re-written. We agree with the reviewer that cfDNA is not a very good marker. On the other hand we have no perfect markers, and cfDNA has been used as a marker in many other studies (as e.g. described in Thålin et al, ref 28). To-day we would perhaps have made another choice but this was what was decided to measure some years ago when the project was planned. We have rewritten the section.

9/ Page 6. There is some controversy about the capacity of cfDNA to activation coagulation- see PMID:27919911. Please see the former point.

10/ Page 6**.** Circulating cancer cells are very rare and it is unlikely that they play a major role in occlusion of vessels. We agree, and we have removed the sentence.

11/ Page 6. The term extracellular vesicles is recommended to describe all forms of vesicles. These can be subdivided into large (microvesicles or microparticles) or small (exosomes) vesicles. This terminology should be introduced. The term EVs, including microvesicles and exosomes, has now been introduced and described in **page 6.** Important references to support the EV description has also been included **(ref 35-36)** page 6**.**

12/ Page 7. The characteristics of the control group should be added to table 1. It is not sufficient to simply describe the median age and range. We have now added this information to Table 1.

13/ Page 7. “first few millilitres were discarded”. Does this mean the first tube? Yes. This has now been corrected (page 7)

14/ Page 9. Ref 28 describes an assay to measure EV TF activity in mice. This should be replaced by PMID:30656275. Hisada Y, Mackman N. Measurement of tissue factor activity in extracellular vesicles from human plasma samples. Res Pract Thromb Haemost. 2019;3(1):44–8- PMID:30656275, has now been added as ref **41 (page 9).**

15/ Page 9. “TF-1” should be “HTF-1”. This has now been revised on page **page 9.**

16/ Page 12. The type of statistical test used for the analysis of the data should be added to the tables. The statistic applied are now included in the Table text for Table 2 and 3.

17/ Page 14. “platelet-poor plasma” should be changed to “PPP”. This has now been revised in **page 14.**

18/ Page 15. It is not clear if the top part of Table 2 shows baseline values for the patients compared with healthy controls or includes all samples? Only baseline values should be used in this comparison. The table includes initial data on controls versus SCLC baseline patients. Moreover, the SCLC patients were further divided into limited (LD) or extended (ED) disease and followed during treatment and follow-up. This has now been clarified in the table. An additional table 2 text now included to clarify this (page 15).

19/ Page 16. The text states that TF activity was significantly increased in the patients compared with the healthy controls. However, Table 2 does not indicated a significant increase. This appears to be an error in the Table. Yes, it was correct that there was 9-fold increase in TF activity, but this was only borderline significant (p=0.51), due to the variation. This has now been explained on page 16.

20/ Page 17**.** The data investigating the effect of adding back isolated vesicles to standard plasma should be removed. The reason for isolating vesicles from plasma is to remove them from the numerous inhibitors that are present in plasma. Adding them back to plasma makes no sense. We agree with the reviewer that they do not describe very exciting results, and especially the results in fig 2A were disappointing. However, we do not agree that it makes no sense to add them to a standard plasma. In this environment, we have “normalized” everything except EVs, and therefore we can compare the effect of these. We have done this before in another population, patients with malignant myeloma (Plos One: [doi.org/10.1371/journal.pone.0210835](https://doi.org/10.1371/journal.pone.0210835)), where we saw a substantial effect. We agree and admit that there is some overlap with the other measurements (TF and PPL), but we think that the results supplement each other. We were surprised that we did not find a difference in TG, because we had found the opposite in the previous paper on malignant myeloma. Since these results also represent a considerable amount of work, we would prefer to keep this figure in the paper. However, if the editor prefers to discard the figure, we are of course willing to do so.

21/ Page 18. A limitation of the study is the small number of thrombotic events (=4). This should be acknowledged. We certainly agree, but this was initially mentioned in the paper (page 22).

22/ Page 20. The Gezelius paper did not have a control group. It did show higher levels of EV TF activity in ED patients compared with LD patients. Yes, it is correct that the Gezelius et al. paper did not include control persons in their study. We apologize for the mistake, but our findings on significantly increased EV TF activity in the LD patients, is comparable. This has been changed in the paper.

23/ Page 20. PMID:21444402 also analyzed TG in cancer patients and should be referenced. We are sorry, but It was not possible to locate the article with the advised PMID number.

24/ Page 21. Do the authors have any evidence that large EVs are lost? No, it was pure speculation. It has now been removed from the article.

25/ Page 21. Doormaal is listed as ref 11 but is not present in the reference list. Thank you for pointing this out. Doormaal is now included as **reference 7**, this has now been rectified in the main text.

26/ Page 21. Zwicker et al ref 13 did not include colorectal cancer patients. Thank you for pointing this out. Cancer patients of different histologies, with and without evidence of acute VTE (venous thromboembolism), were included in this study. This has now been corrected to “Zwicker et al [13] observed a higher concentration of TF positive EVs in cancer patients with VTE compared to the patients without VTE”

27/ Page 21. Contrarily is not a word. According to the Cambridge English dictionary this is an English word meaning “in a way opposite of something”

Reviewer #2: General comments: Language is in part quite vague, especially in introduction.
Can you really declare plasma pooled if it's from one patient? Might not serve as an ideal control due to the high variability of coagulation activity between individuals. Thank you for your comments and suggestions. We have tried to strengthen the language and made several, although smaller changes. We agree with the reviewer’s point regarding “pooled plasma”. It was not a pooled plasma when it originated from just one person, and we have changed it to “Standard plasma”. It can be argued that a real normal plasma from more persons would be preferable, but it is only used as a basis for comparison of EVs from the different patients, and, therefore, we think that it is OK to use this standard plasma as we call it.

Corrections:
104: the activated coagulation factors V and VIII (FVa and FVIIIa, respectively). This has now been corrected (page 6).
109: PPL AND TF activity. This has now been corrected (page 6).
117: patients WERE enrolled This has now been corrected (page 7).
122: OF INR and... This has now been corrected (page 7).
124: technically, ASA and clopidogrel is no anticoagulant therapy. Yes, we agree. This has been corrected to “(platelet inhibitors, ASA and clopidogrel were allowed),” in page 7.
127: Better phrasing: Occurrence of intracranial haemorrhage 3 months prior to start of the study. Thank you, this has now been rephrased, accordingly (page 7).
128: What are effective contraceptives? Thank you for pointing this out, “effective” has been removed from the sentence, and it has been changed to oral contraceptives (page 7).
132: patient files This has now been corrected (page 7).
181: Is thus the right word here? “Thus” has been removed from the sentence (page 9).
287: Table 1. Are T-. N- and M-stage explained somewhere? If not please explain in Figure legend, or leave out if not relevant for the manuscript. Yes, TMN (Tumor, lymph Node, and Metastasis) staging has now been mentioned (and defined) in the result section and in the table text for Table 1.
376 there is no figure 2E We apologize for this fault - this has now been corrected in the figure text ( line 402)
Wouldn’t it make sense to rearrange fig 2 so it’ll fit the order in the text? Yes, we agree. We have changed the text to match the corresponding figures.

Figure legend format is different from fig 1 at least in the pdf received. The legend style for figures 1 and 2 is the same (at least in our Word-paper).

Figure 2a, I get what you’re showing but it’s a little hard to distinguish the traces. Yes, we agree that the various curves are difficult to distinguish because they are not very different. However, these were the attained results, and the impression from the figure is exactly that they are; hardly different, so we hope that it is OK.

6. PLOS authors have the option to publish the peer review history of their article ([what does this mean?](https://eur03.safelinks.protection.outlook.com/?url=https%3A%2F%2Fjournals.plos.org%2Fplosone%2Fs%2Feditorial-and-peer-review-process%23loc-peer-review-history&data=04%7C01%7C%7C22b8cd8b1d524d49f8f208d8e4d8a5f8%7C5968b90c51a64f088b4750ffffbe2e4f%7C0%7C0%7C637510967656778071%7CUnknown%7CTWFpbGZsb3d8eyJWIjoiMC4wLjAwMDAiLCJQIjoiV2luMzIiLCJBTiI6Ik1haWwiLCJXVCI6Mn0%3D%7C1000&sdata=NtVZcaMMz8WeqIGHQSNLNDPji%2BKKRRyFWZTYI6fGjWk%3D&reserved=0)). If published, this will include your full peer review and any attached files.


If you choose “no”, your identity will remain anonymous but your review may still be made public.


**Do you want your identity to be public for this peer review?** For information about this choice, including consent withdrawal, please see our [Privacy Policy](https://eur03.safelinks.protection.outlook.com/?url=https%3A%2F%2Fwww.plos.org%2Fprivacy-policy&data=04%7C01%7C%7C22b8cd8b1d524d49f8f208d8e4d8a5f8%7C5968b90c51a64f088b4750ffffbe2e4f%7C0%7C0%7C637510967656788067%7CUnknown%7CTWFpbGZsb3d8eyJWIjoiMC4wLjAwMDAiLCJQIjoiV2luMzIiLCJBTiI6Ik1haWwiLCJXVCI6Mn0%3D%7C1000&sdata=b%2FYUYWZXIbrhIgwNYOyHZDJi3oV%2BoAhD487hcLEWH2Q%3D&reserved=0).

Reviewer #1: No

Reviewer #2: No

[NOTE: If reviewer comments were submitted as an attachment file, they will be attached to this email and accessible via the submission site. Please log into your account, locate the manuscript record, and check for the action link "View Attachments". If this link does not appear, there are no attachment files.]

While revising your submission, please upload your figure files to the Preflight Analysis and Conversion Engine (PACE) digital diagnostic tool, [https://pacev2.apexcovantage.com/](https://eur03.safelinks.protection.outlook.com/?url=https%3A%2F%2Fpacev2.apexcovantage.com%2F&data=04%7C01%7C%7C22b8cd8b1d524d49f8f208d8e4d8a5f8%7C5968b90c51a64f088b4750ffffbe2e4f%7C0%7C0%7C637510967656788067%7CUnknown%7CTWFpbGZsb3d8eyJWIjoiMC4wLjAwMDAiLCJQIjoiV2luMzIiLCJBTiI6Ik1haWwiLCJXVCI6Mn0%3D%7C1000&sdata=%2FPnAmn4qOpWuL6ugUrO2DQvphwZ5QT%2BUw%2BtFxUEx1dc%3D&reserved=0). PACE helps ensure that figures meet PLOS requirements. To use PACE, you must first register as a user. Registration is free. Then, login and navigate to the UPLOAD tab, where you will find detailed instructions on how to use the tool. If you encounter any issues or have any questions when using PACE, please email PLOS at [figures@plos.org](mailto:figures@plos.org). Please note that Supporting Information files do not need this step.
