# Supplementary material for: Increased activity of procoagulant factors in patients with small cell lung cancer
Source: PLoS One. 2021 Jul 21;16(7):e0253613. doi: 10.1371/journal.pone.0253613 (PMC8294523; doi:10.1371/journal.pone.0253613)
Supplement: Attachment — Submitted filename: Response to reviewers.docx [file pone.0253613.s005.docx]

Dear Editor and Reviewers,

We thank you for the time and effort in reviewing our manuscript. We believe that the final advised revisions and comments has heightened the scientific content and coherence of our article. In the document below, we have addressed and responded to the reviewer’s and editors’ comments - point-by-point. Throughout the manuscript, minor revisions are clarified using track changes mode.

On behalf of all co-authors, we would like to express our appreciation for reviewing our manuscript.

Best regards,

Shona Pedersen, Senior Scientist, Associate Professor

Dep. of Clinical Biochemistry and Clinical Medicine

Aalborg University hospital, Aalborg University

Aalborg, Denmark

**PONE-D-21-04828
Increased activity of procoagulant factors in patients with small cell lung cancer
PLOS ONE**

Dear Dr. Pedersen,

Thank you for submitting your manuscript to PLOS ONE. After careful consideration, we feel that it has merit but does not fully meet PLOS ONE’s publication criteria as it currently stands. Therefore, we invite you to submit a revised version of the manuscript that addresses the points raised during the review process.

Please submit your revised manuscript by Jul 03 2021 11:59PM. If you will need more time than this to complete your revisions, please reply to this message or contact the journal office at plosone@plos.org. When you're ready to submit your revision, log on to https://www.editorialmanager.com/pone/ and select the 'Submissions Needing Revision' folder to locate your manuscript file.

Please include the following items when submitting your revised manuscript:

•A rebuttal letter that responds to each point raised by the academic editor and reviewer(s). You should upload this letter as a separate file labeled 'Response to Reviewers'.

•A marked-up copy of your manuscript that highlights changes made to the original version. You should upload this as a separate file labeled 'Revised Manuscript with Track Changes'.

•An unmarked version of your revised paper without tracked changes. You should upload this as a separate file labeled 'Manuscript'.

If you would like to make changes to your financial disclosure, please include your updated statement in your cover letter. Guidelines for resubmitting your figure files are available below the reviewer comments at the end of this letter.

If applicable, we recommend that you deposit your laboratory protocols in protocols.io to enhance the reproducibility of your results. Protocols.io assigns your protocol its own identifier (DOI) so that it can be cited independently in the future. For instructions see: http://journals.plos.org/plosone/s/submission-guidelines#loc-laboratory-protocols. Additionally, PLOS ONE offers an option for publishing peer-reviewed Lab Protocol articles, which describe protocols hosted on protocols.io. Read more information on sharing protocols at https://plos.org/protocols?utm_medium=editorial-email&utm_source=authorletters&utm_campaign=protocols.

We look forward to receiving your revised manuscript.

Kind regards,

Hugo ten Cate, MD, PhD

Academic Editor

PLOS ONE

Journal Requirements:

Please review your reference list to ensure that it is complete and correct. If you have cited papers that have been retracted, please include the rationale for doing so in the manuscript text, or remove these references and replace them with relevant current references. Any changes to the reference list should be mentioned in the rebuttal letter that accompanies your revised manuscript. If you need to cite a retracted article, indicate the article’s retracted status in the References list and also include a citation and full reference for the retraction notice.

Additional Editor Comments (if provided):

One reviewer still has some relevant suggestions for improvement of the manuscript, the other reviewer abstains from additional comments.

[Note: HTML markup is below. Please do not edit.]

Reviewers' comments:

Reviewer's Responses to Questions

Comments to the Author

1. If the authors have adequately addressed your comments raised in a previous round of review and you feel that this manuscript is now acceptable for publication, you may indicate that here to bypass the “Comments to the Author” section, enter your conflict of interest statement in the “Confidential to Editor” section, and submit your "Accept" recommendation.

Reviewer #2: (No Response)

2. Is the manuscript technically sound, and do the data support the conclusions?

The manuscript must describe a technically sound piece of scientific research with data that supports the conclusions. Experiments must have been conducted rigorously, with appropriate controls, replication, and sample sizes. The conclusions must be drawn appropriately based on the data presented.

Reviewer #2: Yes

3. Has the statistical analysis been performed appropriately and rigorously?

Reviewer #2: Yes

4. Have the authors made all data underlying the findings in their manuscript fully available?

The PLOS Data policy requires authors to make all data underlying the findings described in their manuscript fully available without restriction, with rare exception (please refer to the Data Availability Statement in the manuscript PDF file). The data should be provided as part of the manuscript or its supporting information or deposited to a public repository. For example, in addition to summary statistics, the data points behind means, medians and variance measures should be available. If there are restrictions on publicly sharing data—e.g. participant privacy or use of data from a third party—those must be specified.

Reviewer #2: Yes

5. Is the manuscript presented in an intelligible fashion and written in standard English?

PLOS ONE does not copyedit accepted manuscripts, so the language in submitted articles must be clear, correct, and unambiguous. Any typographical or grammatical errors should be corrected at revision, so please note any specific errors here.

Reviewer #2: Yes

6. Review Comments to the Author

Please use the space provided to explain your answers to the questions above. You may also include additional comments for the author, including concerns about dual publication, research ethics, or publication ethics. (Please upload your review as an attachment if it exceeds 20,000 characters)

Reviewer #2: General comments.

- Replace Standard pooled Plasma (SPP) with SP in figures and methods (‘Isolation of extracellular vesicles’),

Thank you for pointing this out. SPP has now been changed to SP in the actual Figure 2, the associated Figure legend and in the results describing SP (page 17, Line 377).

- The NET paragraph in the introduction contains very bulky language which should be improved, see remarks below.

___________________________

P5 lines 97-98 Better: Neutrophil extracellular traps (NETs) have been linked to the formation of VTEs and may also play a role in cancer-associated thrombosis.

Thank you for rephrasing this sentence. This has now been added to the final manuscript, page 5, line 98.

P6 lines 101-106. . Better something like: Although there is no single marker to determine NETs in plasma, surrogate markers for NETs include plasma levels of cfDNA, citrullinated histone H3 and myeloperoxidase. In addition, cfDNA was described to carry a procoagulant activity on its own and therefore might give insight about NET-associated procoagulant activity in patient plasma samples.

Thank you for a better phrasing on the information on NETs. This has now been added to the final manuscript, page 5, line 100-104.

P6 l. 109 has been = have been

This has now been changed.

P.6 l. 113- 116. Better: A low protC activity was associated with increased mortality in different malignancies, e.g. in non-metastasizing lung cancer (38).

Thank you for rephrasing this sentence. This has now been added to the final manuscript, page 5.

P7 l.127: remove the second 'patients'.

This has now been removed.

P7 l. 131: age over 18 YEARS

This has now been changed from age ≥ 18 to age over 18 years.

P7 l 140: Pregnant and/ or breast-feeding women and women who did not use oral contraceptives were excluded.

Thank you for rephrasing this sentence. This has now been changed in the final manuscript.

P.18/19 Result section Fig 2. Why do you include a curve for Standard Plasma if you don’t mention it in the text? Maybe explain the relevance of the curve not only in the figure legend. Also replace SPP with SP in the Figure and legends.

SPP has been replaced by SP in the figure and the legends (page 18). The thrombograms shown in Fig 2A is included to demonstrate that SP in the absence of EVs indicates longer lagtime with a reduced peak height, whereas controls and SCLC patient samples spiked with EVs, reflected shorter lagtime with higher peak height. We have also included additional text (page 17)

7. PLOS authors have the option to publish the peer review history of their article (what does this mean?). If published, this will include your full peer review and any attached files.

If you choose “no”, your identity will remain anonymous but your review may still be made public.

Do you want your identity to be public for this peer review? For information about this choice, including consent withdrawal, please see our Privacy Policy.

Reviewer #2: No

[NOTE: If reviewer comments were submitted as an attachment file, they will be attached to this email and accessible via the submission site. Please log into your account, locate the manuscript record, and check for the action link "View Attachments". If this link does not appear, there are no attachment files.]

While revising your submission, please upload your figure files to the Preflight Analysis and Conversion Engine (PACE) digital diagnostic tool, https://pacev2.apexcovantage.com/. PACE helps ensure that figures meet PLOS requirements. To use PACE, you must first register as a user. Registration is free. Then, login and navigate to the UPLOAD tab, where you will find detailed instructions on how to use the tool. If you encounter any issues or have any questions when using PACE, please email PLOS at figures@plos.org. Please note that Supporting Information files do not need this step.
